# Structure, magnetic, and photocatalysis of La_0.7_Sr_0.3_MO_3_ (M = Mn, Co, and Fe) perovskite nanoparticles: Novel photocatalytic materials

**DOI:** 10.1007/s11356-023-26411-9

**Published:** 2023-04-13

**Authors:** Mohamed H. Ghozza, Ibrahim S. Yahia, Mai S. A. Hussien

**Affiliations:** 1 Basic Science Department, Marg High Institute of Engineering and Modern Technology, Cairo, Egypt; 2 Nanoscience Laboratory for Environmental and Bio-Medical Applications (NLEBA), Semiconductor Lab., Physics Department, Faculty of Education, Ain Shams University, Roxy, Cairo, 11757 Egypt; 3 Green Research Laboratory (GRL), Faculty of Education, Ain Shams University, Roxy, Cairo, 11757 Egypt; 4 Department of Chemistry, Faculty of Education, Ain Shams University, Roxy, Cairo, 11757 Egypt

**Keywords:** La_0.7_Sr_0.3_MO_3_ (M = Mn, Co, And Fe), Nanoscale perovskites, Sol–gel/auto combustion/co-precipitation methods, XRD/SEM, Hysteresis/magnetization, Photocatalysis process

## Abstract

The present study, La_0.7_Sr_0.3_MO_3_ (M = Mn-, Co-, and Fe-), perovskite, has successfully been synthesized via co-precipitation and sol–gel auto-combustion. XRD, SEM, and EDX characterized the prepared samples. XRD and SEM showed that the as-prepared La_0.7_Sr_0.3_MnO_3_ and La_0.7_Sr_0.3_CoO_3_ have multiphase. La_0.7_Sr_0.3_FeO_3_, in comparison, is nanosized, has a single-phase perovskite, and has a rather homogenous particle size distribution. Additionally, EDX mapping analysis shows that all pieces are distributed uniformly. According to X-ray diffractometer results, all calcined powders contain 100% LSF, more than 15% perovskite phase of LSC, 47% LSM, and other secondary phases, such as cobalt oxide. Aِt room temperature and magnetic field of ± 20 kG, La_0.7_Sr_0.3_MnO_3_ exhibited weak ferromagnetic behavior in a low magnetic field, whereas diamagnetic behavior was seen in a high magnetic field. La_0.7_Sr_0.3_FeO_3_ samples behave as strong ferromagnetic. On the contrary, the photodegradation of La_0.7_Sr_0.3_MnO_3_ is 99% compared to 75% and 91% for other samples under UVC lights of wavelength = 254 nm. The degradation rate for La_0.7_Sr_0.3_MnO_3_ is 0.179 higher, about 3.25 and 2.23, than the other samples. A La_0.7_Sr_0.3_MnO_3_ nanocomposite performs as a photocatalyst to enhance the efficiency of methylene blue photodegradation. This study boosts good UVC photocatalysts with high efficiency for different kinds of dyes. Hence, the catalyst possessed high stability and efficiency for continuous wastewater treatment.

## Introduction

Manganites as perovskite structures with the general formula RAMO_3_, R is the cation for rare earth like La^3+^, Nd^3+^, and Gd^3+^; A is an alkaline cation such as Sr^2+^, Ca^2+^, and Ba^2+^; *M* is a transition metal such as Mn-, Co-, and Fe-; and O^−2^ is oxygen anion. A-site is occupied at corners by rare earth elements or/and alkaline earth metal, B-site is occupied at the center with transition metals (3d, 4d, and 5d), and the oxygen atom is centered at the face (Smitha and Murugendrappa 2019). Due to its unique electrical and magnetic performance, such as ferromagnetic, colossal magnetoresistance (CMR), metal–insulator transitions (*M-I*), and paramagnetic transition (FM–PM), perovskite materials have been studied extensively during these last few years (Sfirloaga et al. 2019). The perovskite family has received considerable attention recently because of its interesting features for potential applications, such as data storage, spintronics, sensors, resistant switching elements, transducers, solid oxide fuel cells, moisture sensing actuators, and catalysts (Verma et al. 2019, Lijak et al. 2016, Tran et al. 2019, Abdel-Latif et al. 2011, Hilal et al. 2012).

Alkyl amine has been demonstrated as the ligand for all-inorganic perovskite nanocrystals with well-defined morphology with considerable attention due to their unique size/shape-dependent optical properties (Feiyan et al. 2020) (Hu et al. 2022) obtained CsPbBr_3_ NRs and showed superior optical performance, demonstrating promising application projections in the photoelectric field. But these practical applications are restricted by poor stability against water, heat, and polar solvents. To avoid such defects, Li et al. (2022) reported CsPbBr_3_@Cs_4_PbBr_6_/SiO_2_ with their double coating structure and found owing stability in a polar solvent (ethanol), water, and heat.

Structures of the perovskites are ideal cubic, but the combination of various ionic radii causes distortion in the structure. These distortions in the octahedral of MnO_6_ can lead to the transition from cubic crystal to orthorhombic, rhombohedral, tetragonal, or hexagonal crystalline phase (Jayakumar et al. 2020, Londoño-Calderón et al. 2018). Several theories have been developed to explain fascinating electrical, magnetic, and photocatalytic properties in several doped manganites, such as double exchange (DE) (Jadli et al. 2021) models between cations, grain boundaries (GBs), Jahn–Teller effect (Li et al. 2019a), and oxygen deficiency (Londoño-Calderón et al. 2018). Partial replacement, La- with divalent alkaline earth elements, Sr-, produces some changes at the A-site and generates mixed M^3+^/M^4+^ valence that causes the ferromagnetic ordering and conductivity of the doping. The average cation radius also influences the tolerance factor, affecting the properties of manganese oxide perovskite (Li et al. 2019b). Relative to the DE model, the metallic characteristics and ferromagnetism are derived from the transfer of roaming electrons, e.g., in the M^4+^ matrix. Manganites’ properties also depend on oxygen deficiency, which might majorly cause expanding the crystal lattice in perovskite ABO_3_ cells (Londoño-Calderón et al. 2018), vacancy, and stress. Currently, the role of B-sites (electron configuration and ionic radius) is also highly considered in manganites; this type of transitional metal causes a density change for the itinerant electrons leading to variations in Curie temperature, magnetization, and conductivity (Ulyanov et al. 2019).

In the present work, the choice of three specific cations, Mn^3+^, Co^3+^, and Fe^3+^, is based on their similar ionic radii due to their adjacent in the periodic table. Recently, various mixed ionic electronics with perovskite structures, including manganite, La_1-x_Sr_x_MnO_3_, La_1-x_Sr_x_CoO_3_, cobaltite, and La_1-x_Sr_x_FeO_3_ orthoferrite, were proposed due to their low price, non-toxicity, capacity, and excellent electrochemical performance (Utami et al. 2019, Abdullah et al. 2020, Ehsani and Raouf 2018, Yang et al. 2019).

When La^3+^ ion is doped by Sr^2+^ ion, an amount of Mn^3+^ (3d^4^, t^3^_2g_↑e^1^_g_↑,:S = 2) ion is replaced by Mn^4+^ (3d^3^, t^3^_2g_↑e^0^_g_↑,:S = 3/2) ion that comes from the motion between two partially filled d shells of an, e.g_.,_ electron. Some factors affect perovskite’s magnetic and photocatalysis properties, such as synthesis route grain size, shape, and density on the particles. Zhou et al. (2018) state that the optimum ratio for the transport property of the system is expected when the ratio of Mn^4+^/ Mn^3+^ is 1:2; this ratio is attained at 33% of the Sr^2+^-doping amount. He reported that the number of manganese ions could also be changed by creating vacancies in oxygen (oxygen deficiency), which can cause modification in the exchange interactions. It also concludes that oxygen deficiency was observed in the vacuum and nitrogen samples. At the same time, the relative concentration of Mn^4+^/Mn^3+^ was reduced, weakening the FM-inflammation interaction between them, resulting in reduced magnetization values. Yousefi and Ranjbar reported that strontium atoms replace atoms of lanthanum in the LSM structure, which are coordinated by twelve oxygen atoms. A Sr^2+^-doping needs to be carefully chosen at between 10 and 30% because a few characteristics, like increasing the coefficient of thermal expansion, can be positively modified (Yousefi and Ranjbar 2017).

Shinde et al. (2011) found that the coercivity of manganite annealed at 900 °C is 26.94 G, attributed to increase grain size. But they explain decreasing coercivity to 10.8 G when the annealing temperature reaches 1200 °C to oxygen deficiency. In the view of the photocatalyst, Ghiasi and Malekzadeh (2014) reported that the photocatalysis activity of La_0.7_Sr_0.3_MnO_3_ nanoparticles in the case of acidic medium or K_2_S_2_O_8_ as an electron accepter was increased. The results, however, showed that the degradation percentage depends more on the particle size and the SSA of samples as a catalyst for photocatalytic activities (Esmaeili et al. 2019). Although ACoO_3_ was discovered in 1950, the cobaltite of perovskite was still attracted by the cobalt’s main aspect, which clearly distinguished the cobalt oxides of transition metal oxides, that is, Co^3+/III^ and Co^IV^ degree of freedom of spin state: the cobalt oxides could possibly be low, intermediate, or high spin state (S = 0, 1, 2 for Co^3+/III^ and S = 1/2, 3/2, 5/2 for Co^IV^). ACoO_3_ cobaltite with 3D corner-sharing network CoO_6_ octahedra often changes from the low spin state (LS, t^6^_2g_ e^0^_g_, S = 0) to the intermediate spin state (IS, t^5^_2g_ e^1^_g_, S = 1) or high spin state (HS, t^4^_2g_ e^2^_g_, S = 2) (Shinde et al. 2012). Moreover, it is possible to achieve oxygen non-stoichiometry by changing preparation methods and the heat treatment temperature. Otherwise, Zhang et al. (2019) reported that Fe(III) compounds are always highly spin, resulting in complex multiples, i.e., a 30% Sr doping in the A-site has resulted in an increase in the oxygen vacancies and enhanced mobility of the oxygen lattice, from Fe^3+^ to Fe^4+^ at the B-site with the proportion of 61.91% and 38.09%, respectively.

While phase purity and particle size depend on preparation methods, several synthesis methods are used, such as solid-state reaction, co-precipitation, sol–gel, microemulsion, frozen-drying, flame hydrolysis, polymeric precursors, hydrothermal synthesis, and citrate-complexation (Sfirloaga et al. 2019). For example, a solid-state reaction method occurs at high temperatures (> 1000 °C), which results in non-porous, irregular powder products, uncontrolled particle size, and low surface area (Kaewpanha et al. 2019). Other interesting chemical approaches in low sintering temperature and short reaction time (Li et al. 2019b), such as the sol–gel process and co-precipitation, have been reported to prepare materials with improved control of grain size and uniform particle size distribution and morphology (Hannora and Hanna 2019, Ghozza et al. 2013, Shlapa et al. 2018). Therefore, by the co-precipitation method, we focus on preparing La_0.7_Sr_0.3_MnO_3_ and La_0.7_Sr_0.3_CoO_3_, but the sol–gel auto-combustion route to prepare La_0.7_Sr_0.3_FeO_3_ samples.

Organic and toxic pollutants pollute water sources in different dyeing factories (Sakamoto et al. 2019). Dyes are problematic because the chemical families that produce good dyes also cause human toxicity and are regarded as fatal poisons (Qu et al. 2014). The most serious problem in developing countries is environmental pollution from dye wastewater. Common methods of treatment dyes with a complex aromatic structure can be very difficult to degrade since products resulting from different reactions, such as hydrolysis and oxidation in aqueous media, produce toxicity and carcinogenic substances. These products should be removed with appropriate treatment methods for public health and safety (Gowthami et al. 2018). In recent years, enormous research and development have gained considerable importance as an environmental remediation process in photocatalysis because it can fully remove organic and inorganic toxins from water pollutants by using nanosized metal oxides as catalysts (Yerkinova et al. 2018; Kanakaraju and Wong 2018; Guillaume et al. 2018). Several metal oxide nanosized semiconductor nanoparticles have been used in different applications, including dye solution photodegradation and contaminated water. The predominant research in the photocatalysis field for wastewater purification has included semiconductors of wide bandgap and high surface-to-volume ratio material such as titanium dioxide (TiO_2_), zinc oxide (ZnO), tin oxide (SnO_2_), and zirconium oxide (ZrO_2_) (Anandan et al. 2020). This compound is called the hole-doped. That hole doping plays a role in transferring the electronic system to the Fermi level to ensure that conductivity and valence band behavior is observed. The coexistence of electrons and holes in manganites introduces them into optical systems as promising materials (Esmaeili et al. 2019). Chahar et al. (2021) observed that the ferrite’s degradation was enhanced by an increasing cobalt level with a maximum degradation efficiency (77%) for *x* = 0.5. The lowest degradation efficiency (~ 65%) for *x* = 0.0 per 1 h was achieved by visible light irradiation. However, due to their unique electronic properties and crystal structures, many perovskite oxides have visible light photocatalytic activity (Pena and Fierro 2001a, b). Doped alkaline rare earth transition metal perovskite-like structure oxides reduce the bandgap energy values because this feature increases the charge carrier separation (photogenerated electrons and holes) (Ismail et al. 2010). Intense studies were conducted on the materials because their electrical and optical properties can be tuned, indicating control of the rational design structure in ABO_3_ perovskite by cationic substitutions (Ismail et al. 2009; Shui et al. 2000). So, we can say that perovskite compounds are one of the promising structures that adapt the bandgap values to collect the visible-light absorption and the capacity of the band edge to meet the specific photocatalysis needs. Abdel-Latif et al. (2017) say that Nd_0.6_Sr_0.4_MnO_3_ has a small bandgap of 2 to 2.98 eV, which can be controlled by changing the annealing temperature of its rings. The researchers investigated the photocatalysis efficiency of the annealed Nd_0.6_Sr_0.4_MnO_3_ perovskite under visible light and found that annealed perovskite at 500 °C was a better photocatalyst than that of 800, 1000, and 1150 °C. However, little work has been used perovskite nanoparticles as a catalyst. Previous works have not comprehensively considered the synthesis route and transition metal spin role in the magnetic and photocatalysis process.

## Experimental technique

### Synthesis of La_0.7_Sr_0.3_MnO_3_, La_0.7_Sr_0.3_CoO_3_, and La_0.7_Sr_0.3_FeO_3_ nano-perovskites 

Nano-perovskites La_0.7_Sr_0.3_MnO_3_ and La_0.7_Sr_0.3_CoO_3_ (hereafter will be called LSM and LSC, respectively) were prepared by a co-precipitation route. According to Eqs. 1 and 2, the stoichiometric quantity of lanthanum nitrate-hexahydrate (La(No_3_)_3_.6H_2_O), strontium chloride hexahydrate (SrCl_2_.6H_2_O), manganese sulfate (MnSO_4_), and cobalt chloride hexahydrate (CoCl_2_. 6H_2_O) was weighed by a digital balance and dissolved separately in 50 mL distilled water afterward; the solution was put on a hot-stirring plate at 600 rpm and 60 °C for 1 h until complete dissolution occurred. The solutions were mixed in one beaker and on magnetic stirring again; then, 100 mL of CTAB (cetyltrimethylammonium bromide) was added to prevent particle aggregation. To avoid the NO_x_ gases, the solution moves to an isolated position and adds NH_4_OH carefully as a reaction medium to reach a pH = 10. Finally, this solution was heated and stirred under the same conditions until a precipitate was formed. The residue was washed several times with filtration paper and dried in the furnace at 80 °C overnight.

The ultimate products were sintered at 1000 °C for 10 h in the air to obtain the perovskite phase. La_0.7_Sr_0.3_FeO_3_ sample (hereafter will be called LSF) was prepared with a low cost and easily modified sol–gel/auto-combustion process based on an easy precursor. High-purity lanthanum nitrate (LaN_3_O_9_.6H_2_O), strontium nitrate (Sr(NO_3_)_2_), and iron nitrate (Fe(NO_3_)_3_.9H_2_O) as starting materials were taken in appropriate stoichiometric ratio. The precursor solution was prepared by fully dissolving the constituents in a beaker in distilled water (precursor/starter materials). As a chelating agent, a specific amount of citric acid (C_6_H_8_O_7_·H_2_O) is dissolved in distilled water in a separate beaker. The molar ratio of citric acid to La_0.7_Sr_0.3_FeO_3_ was 1:1. The dissolution was carried out by magnetic stirring at 600 rpm. The nitrates, citric acid, and NH_4_OH (to control pH at 9) were put together immediately into a third big beaker. The solution was mixed at 80 °C using a magnetic stirrer for 1 h until a clean and transparent solution was found, while some ethylene glycol was added. The homogeneous precursor solution was then dried at 120 °C for 24 h to remove the water content. The powder is put in the beaker on a hot plate at a temperature of 250 °C until instantly combustion occurs and continues for 30 s. The obtained powder was calcined at 1000 °C for 6 h. The obtained powder was pressed at 10 tons into pellets of a diameter of 13 mm and thickness of 2 mm with a stainless steel dye mold to calculate the density. 

### Devices and measurements

#### XRD and SEM measurements

The X-ray diffractometer Bruker D8 Advance checked the sample structure with a *CuK*_*α*_ radiation (*λ* = 1.5406 Å) system. The data was collected in step-scanning mode within the *2θ* range of 20–80° for LSM and LSC and 10–80° for LSF with a step size of 0.02°. An XRD diffraction pattern investigated the samples’ phase identification and structural analysis. The XRD peaks were indexed using the open crystallography database (COD) and X’Pert high score software program. The XRD pattern analysis used Fullprof software to characterize the crystalline structure. The surface morphology of the three annealed at 1000 °C LSMO and coated with gold for 4 min samples were observed using a scanning electron microscope (SEM, Vega 3 SBU Tescan, Czech Republic instrument operated at 30 kV) equipped with an energy dispersive spectrometer (EDX).

#### Magnetic measurement

Magnetization was measured at the room temperature of three samples using a vibrating VSM (Lake Shore 7410) magnetometer in the magnetic field range of ± 20 kG.

#### Photocatalytic activity

The photocatalytic activity of La_0.7_Sr_0.3_MO_3_ was inspected using MB dye (MB, C_37_H_27_N_3_Na_2_S_3_O_9_) under UVC- radiation for the model pollutant using a wooden photoreactor at room temperature, designed by I.S. Yahia and his group at NLEBA/ASU/Egypt; more details about the photoreactor are mentioned in Hussien et al. (2020a, b**)**. The 5 mg catalyst and 100 mL dye solution (10 mg/L) were mixed using a magnetic stirrer for about 30 min to achieve the La_0.7_Sr_0.3_MO_3_ adsorption/MB balance between the photocatalyst and the MB. The suspension was collected by the irradiation process at 1 mL every 5 min from the mixture. The mixture was then irradiated with UVC lamps of 18 watts of wavelength = 254 nm. For analysis, a solution of 1 mL was obtained and filtered inside the quartz cuvettes for the absorbance measurements versus the incident wavelength in the range from 400 to 800 nm using a UV–Vis Double Beam Spectrophotometer. The reusability and stability of the synthesized samples were monitored repeatedly for 5 cycles.

## Result and discussion

### X-ray analysis of La_0.7_Sr_0.3_MO_3_, nano-perovskites 

**Fig. 1:**
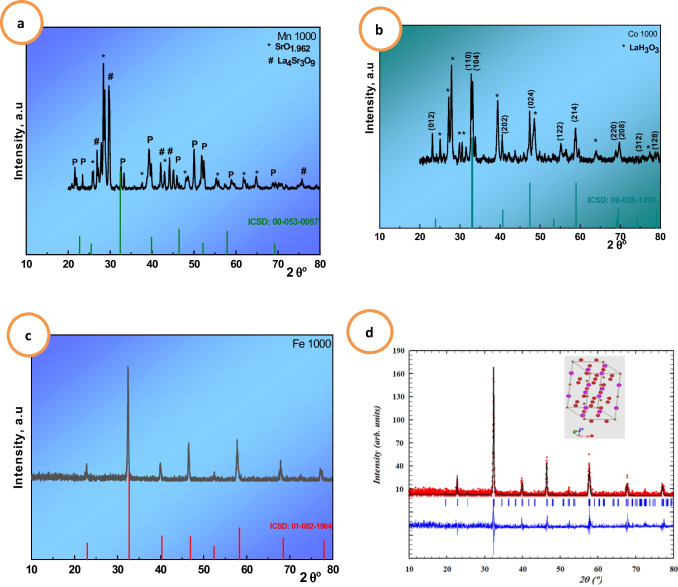
**a–d** XRD patterns for LSM (**a**), LSC (**b**), and LSF (**c**) samples sintering at 1000 °C. **d** Fullprof refinement for Fe-perovskite sample; the inset is perovskite structure

Figure 1a shows the XRD pattern for the La_0.7_Sr_0.3_MnO_3_ sample sintering at 1000 °C. The LMS sample reveals a little perovskite phase ratio of about 15%, belonging to ICSD# 00–053-0075 (Q) according to the X’pert high score program besides SrO_1.962_ and La_4_Sr_3_O_9_ as secondary phases. The compound is crystallized in an orthorhombic structure with the symmetry of the Pcmn space group. LSC sample sintering at 1000 °C shows a perovskite phase with a ratio of 47.5% belonging to ICSD# 00–36-1393 (Q), according to X’pert high score program having Rhombohedra structure system with space group R-3C (167) beside one secondary phase of LaH_3_O_3_ ICSD# 42–0343 (D). LSF sample is indexed as Rhombohedra with 100% perovskite phase structure with space group R-3C (167) ICSD# 01–082-1964. The variation in the perovskite forming ratio may be due to the difference in the synthesis route. It indicates that the sol–gel/auto-combustion method saves time and energy and is better in crystalline phase forming. The lattice parameters and volume of the unit cell are represented in Table 3.

**Fig. 2:**
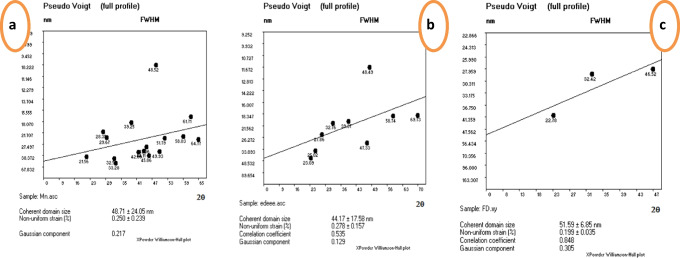
**a** The LSM, **b** LSC, and **c** LSF crystal size by William son and Hamilton fitting using pseudo-Voigt model

Because of the multiphase, performing refinement for LSM and LSC samples is difficult. Rietveld refinement analysis was carried out for the LSF sample. The background is refined by using the pseudo-Voigt function with the 12-coefficients Fourier-cosine series and other parameters such as scale factor, sample displacement, isotropic thermal parameter *B*_iso_, and peak asymmetry shown in Fig. 1d. The inset in Fig. 1d is the structure of LSF perovskite. The compound is crystallized into an R-3C symmetry rhombohedral structure. The small value of the fitness indicator *χ*^2^ confirmed the perfect quality of the refining. The Wyckoff equivalent positions in the cell follow: (La, Sr) at Fig. 2a (0, 0, ¼), (Fe) at Fig. 2b (0, 0, 0), and O at 18e (x, 0, ¼). The lattice parameters, unit cell volume (V), value of goodness of fit *χ*^2^, and reliability factors (R%) are listed in Table 1. To check the structure stability, Goldschmidt tolerance factor, *t*, Eq. 1 is used and calculated using ionic radii from Shannon (1976) as La^+3^ = 1.36, Sr^+2^ = 1.44, Mn^+3^ = 0.645, Mn^+4^ = 0.56, Co^+3^ = 0.61, Co^+4^ = 0.53, Fe^3+^ = 0.645, Fe^4+^  = 0.585, and O^−2^ = 1.4 Å. The calculated values have proven little distortion perovskite structure and are between 0.97 and 0.99 as shown in Table 1 as

**Table 1:** The structure parameters of the studied samples

Parameter	LSM	LSC	LSF
Perovskite phase (%)	15	47	100
Structure (SG)	Orth., (Pcmn)	Rhom., (R-3 m)	Rhom., (R-3 m)
a (Å)	5.8130	5.3972	5.4652
b (Å)	5.5110	5.3972	5.4652
c (Å)	7.5245	13.336	13.3864
V (Å^3^)	243.1618	336.892	346.215
Cs, Sch (nm)	32.3	38.3	44.2
CS, W–H (nm)	48.7	50.8	51.59
D (nm)	214.5	190	174.2
*n* = D/C_S_	4.76	3.91	3.26
ρ_XRD_ (g.cm^−3^)	6.58	6.69	5.92
ρ_cal._ (g.cm^−3^)	4.6101	4.0118	4.9363
Porosity (%)	30	40	22
SSA (m^2^.g^−1^)	4.25	4.72	5.82
*t*	0.9792	0.9912	0.9711
Dislocation × 10^−4^	3.36	4.30	12.8
Strain × 10^−4^	6.00	7.13	11.1
χ^2^	**-**	**-**	5.68
*R*_wp%_	**-**	**-**	5.72
*R*_p_	**-**	**-**	5.2
*R*_F_	**-**	**-**	3.3

1 $$t=\frac{\left[\left(1-y\right){r}_{{A}^{3+}}+\left(y\right){r}_{{A}^{2+}}\right]+{r}_{O}}{\sqrt{2} [\left({\left(1-x\right)r}_{{B}^{3+}}+\left(x\right){r}_{{B}^{4+}}\right)+{r}_{O}]},$$

**Fig. 3:**
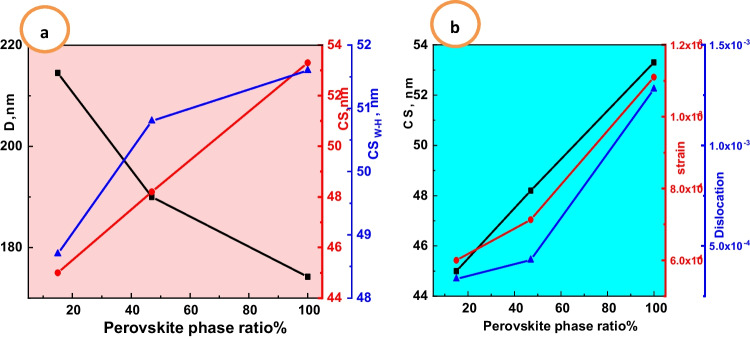
**a** The perovskite phase ratio formed vs. crystal size from Scherrer’s (CS) and W–H (CS_W-H_) formula and grain size (D). **b** The perovskite phase ratio formed vs. crystal size from Scherer (CS) and lattice strain and dislocation density

Figure 3a reveals the most intense peak crystallite size (CS) according to Scherer’s formula with Eq. 2 as well as William’s son and Hamilton (CS_W-H_) having formula with Eq. 3 by XPowder software computer’s program (Ghozza et al. 2020) vs. perovskite ratio. The behavior of CS and CS_W-H_ increases with increasing perovskite ratio, contrary to particle size due to secondary phases. The crystallite size estimated using Scherer’s equation is found to be 32.3, 38.3, and 44.2 nm for compositions LSM, LSC, and LSF, respectively. At the same time, CS_W-H_ has the same trend that decreases with increasing perovskite phase formed for compositions LSM, LSC, and LSF are 48.7, 50.8, and 51.6 nm, respectively. Figure 3b reveals the linear dependence of the crystallite size (CS), dislocation, and strain lattice on the perovskite ratio as

2 $$D=\frac{0.9\lambda}{\beta\;\cos\;\theta},$$

3 $$\beta\;\cos\;\theta=\frac{0.9\lambda}D+4\varepsilon\;\sin\;\theta,$$

The calculated density of the La_0.7_Sr_0.3_MO_3_ uses normal relation *ρ*_cal_. = m/V, where *m* is the sample’s mass in g and *v* = *π*^*2*^*rd*. The porosity of the pellets was calculated by Eq. 4 (da Conceição et al. 2011):

4 $$P=\frac{{\rho }_{XRD}-{\rho }_{cal}}{{\rho }_{XRD}}\times 100,$$

The whole structure parameters, measured and calculated, are represented in Table 1. The porosity is estimated to be 30%, 40%, and 22% for LSM, LSC, and LSF samples; these values are less than those obtained in other published (da Conceição et al. 2009) (see Table 2).

**Table 2:** The theoretical and calculated weight percent of La_0.7_Sr_0.3_MO_3_

Samples	Theoretical molecular weight	La, (%)	Sr, (%)	M, (%)	O, (%)
LSM	226.4540	42.93	11.60	24.26	21.19
LSC	230.4497	42.19	11.40	25.57	20.82
LSF	227.3617	42.76	11.56	24.56	21.11

### Microstructure and surface morphology

**Fig. 4:**
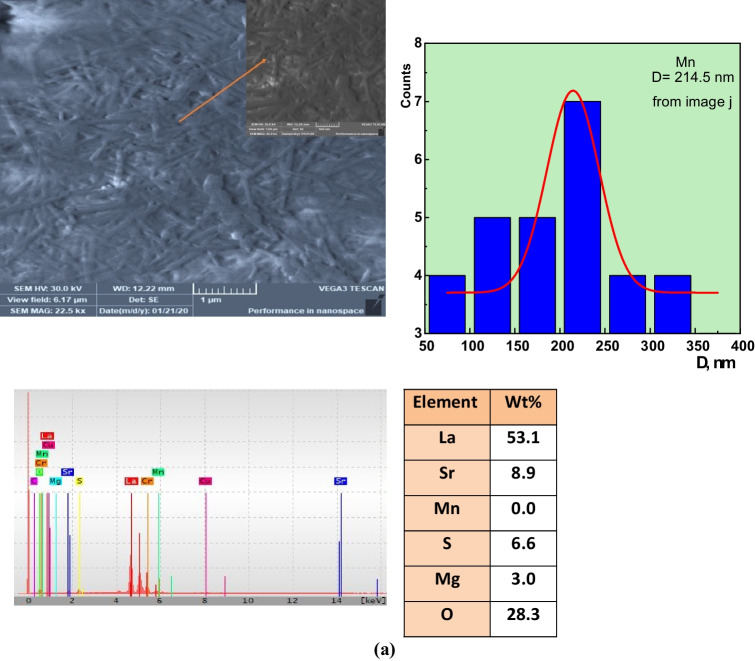

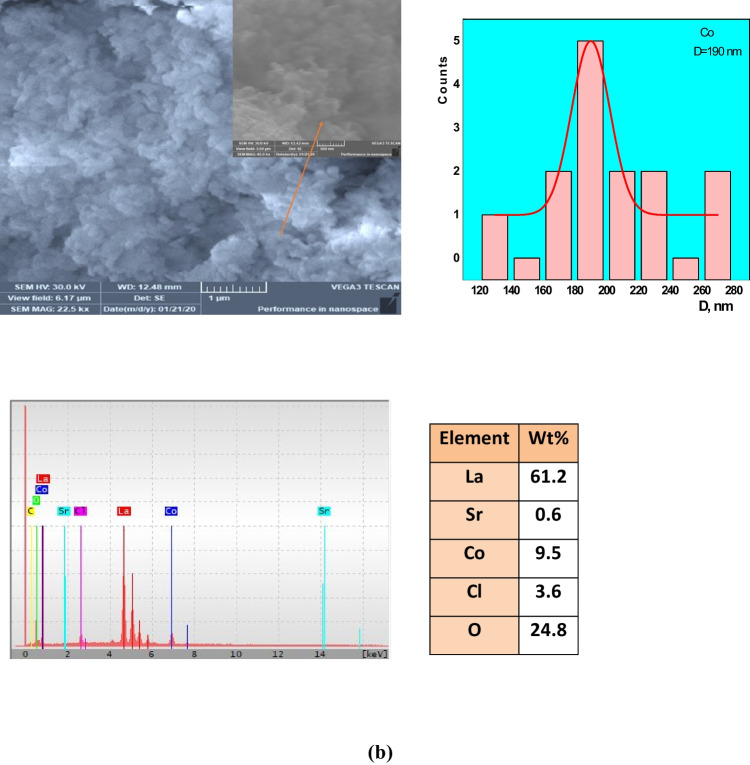

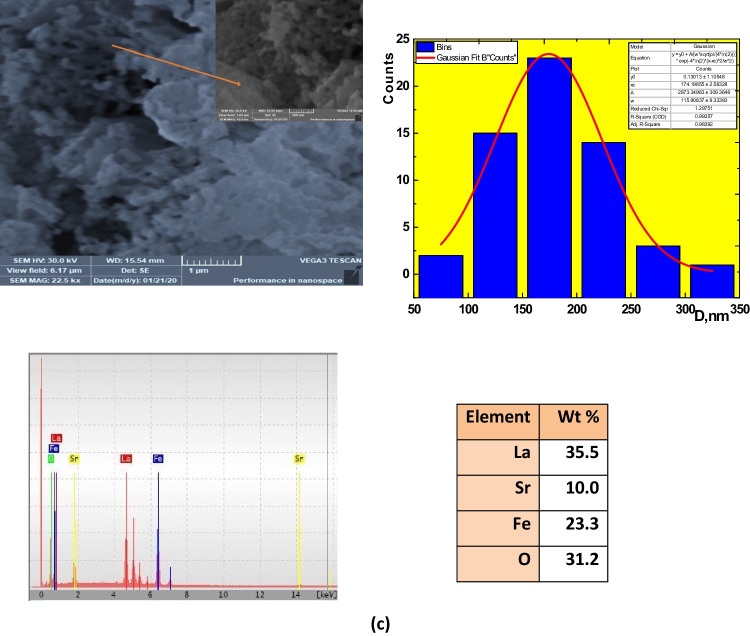
SEM, particle size distribution, EDX, and wt% of **a** LSM, **b** LSC, and **c** LSF

The surface morphology in the three samples has not changed dramatically (see Fig. 4a–f). The three samples were similar in mean grain sizes. These images indicate that particles in each sample are well-resolved and distinctive. For LSM specimens, grain agglomeration increased, intergranular gaps increased, and the stepped morphology increased, which agreed with the literature. These morphological aspects are due to whether the oxygen-deficient in the sample, the escape of Mn- cation from the sample, and the presence of some impurities such as Mg- and S- elements as contamination during the preparation process. In addition, the particles are irregular in form and quite uniform, particularly the Fe- sample, due to the different preparation methods that agree with previous work (Wiglusz et al. 2015; Xu et al. 2008). The diameters of the particles are determined using the ImageJ software program by Gaussian fit of the total partitions. With the largest ionic radius of rare earth ion doping, the average particle size increases, and the samples of LSM, LSC, and LSF showed 214.5, 190, and 174.2 nm, respectively. The variance between the calculated crystallite size by Scherrer’s and W–H formula may be due to the chosen peak. Some perovskite-formed peaks for calculating CS and CSW-H are 15%, 47%, and 100% for LSM, LSC, and LSF samples. But grain size, *D*, considers agglomerates of various crystallite size of different phases. The indicator of agglomeration *n* is 4.76, 3.91, and 3.26 for LSM, LSC, and LSF samples, respectively.

Additionally, the chemical constitution of the samples has been checked by EDX analysis in Fig. 4a–c to examine the possibility of cation non-stoichiometry along different preparation routes. The relative atomic percentage of sampled elements of La-, Sr-, M-, and O- has been shown to be roughly unsatisfactory in the measurement, confirming the presence of certain accumulations and the lack of cations sensitive to the synthesized method variations. The best explanation for that percentage discrepancy is that EDX can only give you the percentage composition of the selected region you map in your analysis, which may vary when you choose another position on your sample. However, the parameter of the specific surface area, SSA, is extremely determined by the following Eq. 5, since photoreaction occurs on the surface area (Esmaeili et al. 2019):

5 $$\mathrm{SSA}=\frac{6\times 1000}{\uprho \times \mathrm{D}},$$

where $\rho$ is density from XRD and *D* grain size. Indeed, the presence of more reactive sites is more likely because of the smaller particles. This means that the smaller the size, the bigger the SSA. The calculated SSA is 4.25, 4.72, and 5.82 for LSM, LSC, and LSF samples, respectively. Berger et al. (2009) obtained the same results.

### Magnetic properties

**Fig. 5:**
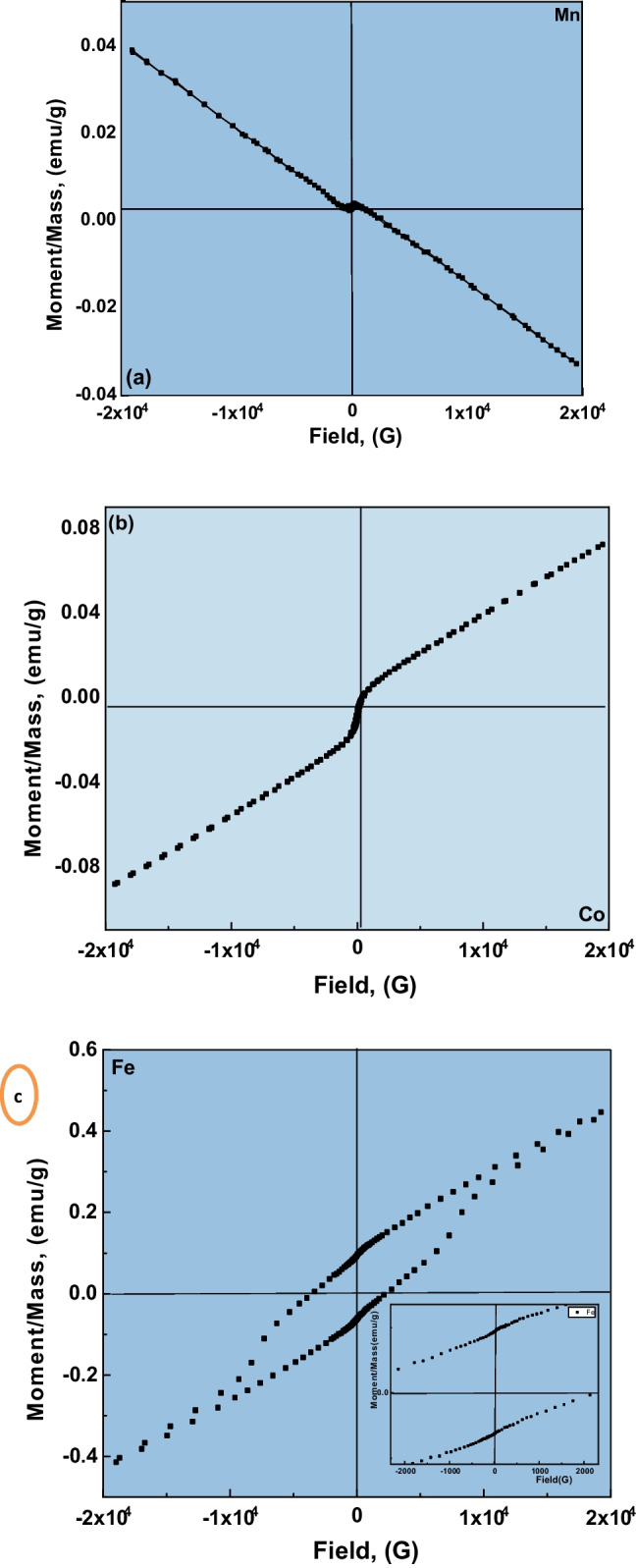
The M-H hysteresis loops of **a** LSM, **b** LSC, and **c** LSF perovskite samples

Figure 5a–c display the M–H curves of LSM, LSC, and LSF perovskite samples under the applied magnetic field of ± 20 kG at room temperature. The corresponding saturation magnetizations (*M*_s_), coercivity (*H*_c_), retentivity (*M*_r_), and squareness (*S*) are listed in Table 3. The *M-H* hysteresis loop of the LSM powder exhibited two distinct behaviors. Uncommon weak-ferromagnetic-like behaviors at the low applied magnetic field, ± 1000 G, with saturation magnetization and coercivity values, are 0.0359 emu/g and 133.78 G, respectively. Whereas increasing the applied magnetic field was nearly above ± 1500 G, the magnetization increased in the direction opposite to the externally applied magnetic field (anti-S shaped), demonstrating the intrinsic diamagnetic behavior of LSM. Such behavior is shown by Dong et al. (2020). The uncommon ferromagnetic performance of the polycrystalline LSM in the low magnetic field is likely to take place due to the existence of some potential point defects as vacancies that might form at the surface or near-surface region in the polycrystalline sample during synthesis (Handal et al. 2019); the existence of secondary phases beside perovskite phase confirms that opinion. Furthermore, these unusual behaviors are ascribed to particles’ surfaces which might exhibit uncompensated spins that were ferromagnetically ordered and are missing in the bulk counterpart (Handal et al. 2019). Similar notes were reported for nanosized oxides due to the origin of ferromagnetism in near-surface regions where the thermal treatment could significantly reduce their density (Qin et al. 2010). In addition, such observation could be attributed to the bulk (Jayakumar et al. 2020) LSM samples, where a particle size of 214.5 nm and a great aggregation indicator factor of 4.76 are represented in Table 2.

**Table 3:** Magnetization parameters of the studied materials

Samples	Coercivity (*H*_c_), G	Magnetization (*M*_s_), emu/g	Retentivity (*M*_r_) × 10^−3^G	Squareness
LSM	133.28	0.0359	0.196	5.473
LSC	59.747	0.0800	1.799	0.022
LSF	2905.4	0.4301	77.98	0.181

The saturation magnetization increases with a change in the transition ion from LSM (*M*_*s*_ = 0.0359 emu/g) to good agreement with *M*_*s*_ = 0.0552 emu/g (Jayakumar et al. 2020), LSC (*M*_*s*_ = 0.0800 emu/g) to LSF (*M*_*s*_ = 0.4301 emu/g). LSC samples exhibit paramagnetic behavior. LSF showed a clear hysteresis loop that indicates ferromagnetic behavior over the whole magnetic field at room temperature. Furthermore, the squareness ratio (SQR) of LSF is quite large compared to the two other samples. The large SQR value indicates that the homogeneity of the particle (Nguyen and LeMinh 2012) is predominant in the LSF sample and confirms the ferromagnetic behavior. Other authors (Handal et al. 2019) reported a peculiar ferromagnetic behavior for the perovskite. The ferromagnetic behavior was interpreted by the presence of F-centers created by oxygen vacancy—the F-center exchange coupling (FCE). With the FCE mechanism, a magnetic ion such as TM or RE is trapped in the form of oxygen vacancies which act as a coupling center. The bounding magnetic polaron (BMP) model is also used to explain the ferromagnetism of this type (Handal et al. 2019). The interplay of the connected carrier with the magnetic ions under its surface (donor or acceptor such as vacancies) results in the polar magnet bound (BMP) formation. Ferromagnetic interaction is caused when two polarons overlap. When the polarons overlap largely and this type can remain at high temperatures, strong ferromagnetic interactions are caused.

For the LSM sample, the weak ferromagnetic property in a low magnetic field can reasonably be explained in a slightly canted spin arrangement (Zhang and Jianhua 2010). In comparison, diamagnetic behavior was seen in a high magnetic field due to the presence of impurity phases, which may be prevented the magnetic domain rotation in the direction of the magnetic field from the low values of the coercive field. The magnetization versus magnetic field shows a small coercive field and an unsaturated magnetization, indicating that some LSM sample nanoparticles are blocked at room temperature (Rostamnejadi et al. 2009). Abdel-Khalek et al. (2009) explain that the decrease of coercive field can be attributed to the increase of the grain size for the polycrystalline ferromagnets, and grain boundaries are magnetic domain boundaries. Additionally, the coercive field decreases with increasing grain size as follows: *H*_c_ = *H*_c*,*o_ + (*K*_M_*/D*) where *H*_c*,*o_ reflect the coercivity due to other effects, such as internal stresses and impurities, *K*_M_ is a constant, and *D* is the grain size.

### Photocatalytic process

**Fig. 6:**
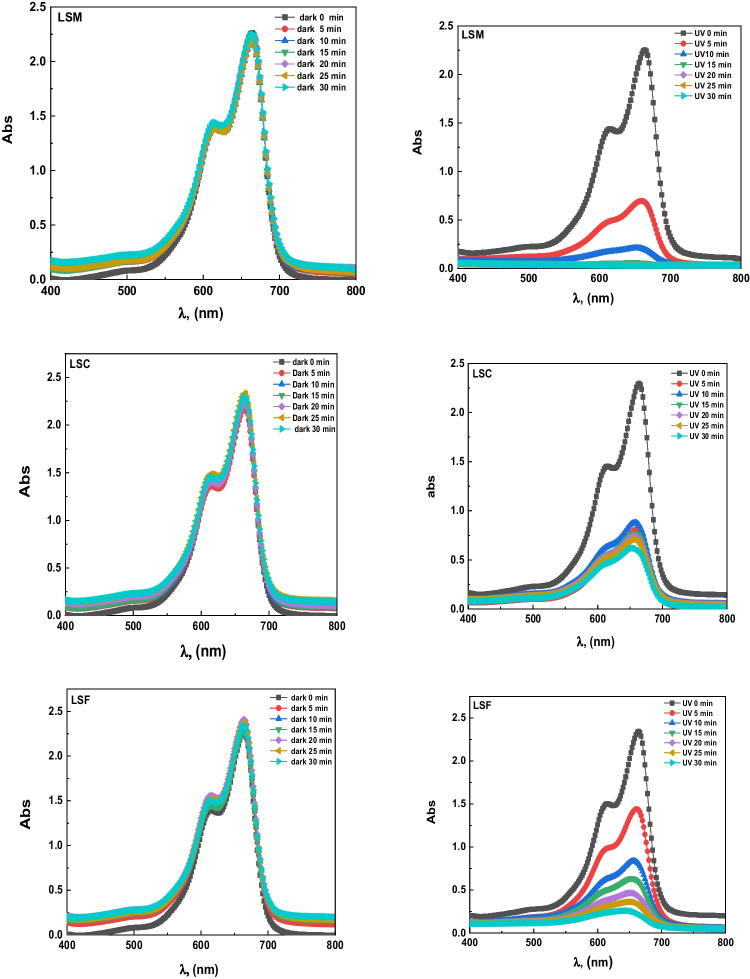
The absorbance vs. wavelength under dark and UVC- lights using MB dye for the studied samples (**a** LSM, **b** LSC, and **c** LSF)

**Fig. 7:**
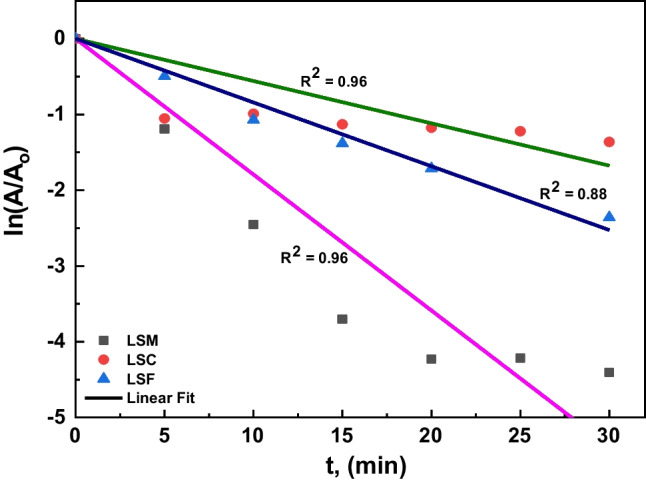
The ln(*A*/*A*_o_) vs. time and fitting for **a** LSM, **b** LSC, and **c** LSF samples

**Fig. 8:**
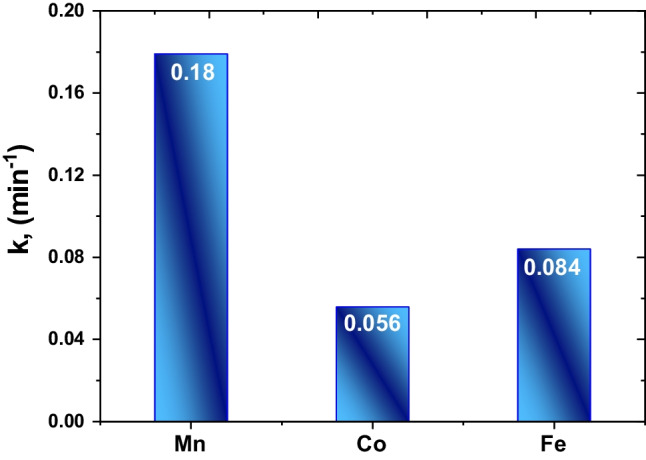
The degradation rate (*k*) for LSM, LSC, and LSF samples

**Fig. 9:**
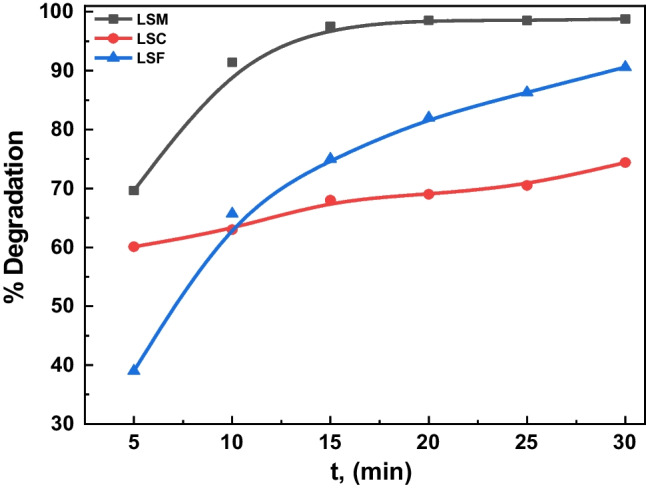
The percentage of degradation vs. time for **a** LSM, **b** LSC, and **c** LSF samples

The photocatalytic absorbance of MB of the different catalysts (blank, LSM, LSC, and LSF) at room temperature in dark and UVC- light is shown in Fig. 6a–c, respectively; for three samples, the maximum absorbance reveals at 664 nm wavelength. The MB is adsorbed over the La_0.7_Sr_0.3_MO_3_, and an adsorption–desorption balance is reached after 30 min during the photocatalytic treatment (see Fig. 7); the kinetics and rate constants were analyzed and computed with the help of Eq. 6 (Hussien et al. 2020a, b):

6 $$\ln\;(A/A_o)=-kt,$$

where *A*_o_ is the initial absorbance, *A* is absorbance after time *t*, and *k* is rate constant; the plot of ln(*A/A*_o_) against the time *t* is linear according to Eq. 6. Thus, the value of *k* can be obtained directly via its slope (see Fig. 7) to be 0.179, 0.055, and 0.08 for LSM, LSC, and LSF, respectively. These results agree well with another catalyst, as mentioned in Table 4. Figure 8 plots the *k* values for the studied three samples. The following equation calculated the efficiency of the photocatalytic degradation process of the studied samples (Hassan et al. 2022):

7 $$\%\ Degradation=\frac{{A}_{0}-A}{{A}_{0}}\times 100,$$

where *A*_o_ is the initial MB solution absorbance without any pre-degradation exposure and *A* is the solution MB absorbance after photo-irradiation for time *t*. MB absorption was estimated at 664 nm of peak wavelength, the maximum absorption for MB taken in the UVC- spectrum at first and every 5 min. A degradation of MB dye under UVC- light irradiation determined the photocatalytic activities of candidate materials. The degradation percentage of LSM, LSC, and LSF for 30 min is 99%, 78%, and 95.5%, respectively, as shown in Fig. 9.

**Table 4:** The calculated photocatalysis parameters of the studied samples

Samples/photocatalyst parameters	Dye	Synthesized method	Dye conc.	Catalyst conc.	% deg	*K (min*^*−1*^*)*	Light source	Refs.
LSM	MB	Co-precipitation	0.01 g/L	10 mg	99	0.179	UVC-light	Present work
LSC	MB	Co-precipitation	0.01 g/L	10 mg	75	0.056	UVC-light	
LSF	MB	Auto-combustion	0.01 g/L	10 mg	91	0.084	UVC-light	
La_0.7_Sr_0.3_MnO_3_	MO	Citrate method	5 ppm	25 mg	96	0.179	Visible	[21]
Nd_0.6_Sr_0.4_MnO_3_	MB	Sol–gel	-	-	100	-	Visible	[42]
La_0.7_Ba_0.3_MnO_3_	MO	Microwave irradiation	10 ppm	0.005 gr	81.97	0.0259	Visible	[36]
TiO_2_/g-C_3_N_4_	MB	Combustion	20 mg/L	30 mg	92	0.014	Visible	[58]

As well known, OH^•^ radicals degrade dye molecules into simpler compounds (CO_2_ and H_2_O). The impact of irradiation time on the percentage of dye degradation can be seen in Fig. 7b. From the curve, the amount of degraded OH• radicals increases with increasing irradiation time (Rahmayeni et al. 2020). The photo-degradation originated when the UVC- light irradiated the catalyst, so the photodegradation mechanism of dye solutions is based on the redox reactions for perovskite samples. The photon energy (*hυ*) absorbed is equal to/or greater than the perovskite energy gap. During the photoexcitation process, the valence band (VB) electron jumps to the conduction band (CB) and lets h^+^ on VB, which reacts with the adsorbed ^−^OH ions or the H_2_O on the perovskite surface, producing OH^•^.

On the other hand, the electron reduces the amount of O_2_. On the other hand, O_2_• increases the number of other oxidative O_2_ (i.e., OH^•^ and H_2_O_2_). It takes the oxidation portion and prevents e^−^/h^+^ pair recombination at VB. The protonation of superoxides produced peroxides, and the formation of OH^•^ radicals was dissociated. Conversely, h^+^ is responsible for producing reactivity species OH^•^ radicals using the VB hydroxyl ions. The radicals manufactured on the surface of radiated La_0.7_Sr_0.3_MO_3_ and OH^•^ are very strong agents that attack and cause the dye molecules to become mineralized as simple molecules (CO_2_ and H_2_O) (Rahmayeni et al. 2020). It is clear from the photocatalytic process of the LSM sample, with a crystalline size of 48.7 nm, that the mixed structure La_0.7_Sr_0.3_MnO_3_ (15% orthorhombic” and 85.82% impurities) is a superior photocatalyst candidate than that of two other samples having the mixed structure (47% rhombohedral and 53% secondary phases). The observed photodegradation was 99% in the LSM sample, and the photodegradation rate of the sample LSM (0.179 min^−1^) is significantly about 3.25–2.23 times higher than that of the LSC and LSF samples, respectively, which is good agreement with the previous work (Abdel-Latif et al. 2017). These results quite agree with the obtained results by Ghiasi and Malekzadeh (2014) and Hussien and Yahia (2021). The superiority of the La_0.7_Sr_0.3_MnO_3_ can be attributed to an irregular crystallographic structure, with the Mn–O-polyhedral distortion of the secondary phases. This work showed that the high UVC- light uptake, lattice distortion, and narrow bandgap are key factors for the high photocatalytic activity of the obtained lanthanum strontium-doped manganite.

Furthermore, the degradation rate for La_0.7_Sr_0.3_MnO_3_ is 0.179 higher, about 3.25 and 2.23, than the other samples. A La_0.7_Sr_0.3_MnO_3_ nanocomposite performs as a photocatalyst to enhance the efficiency of methylene blue photodegradation owing to some structural, morphological, and electronic configuring reasons. The structural reason involved the presence of SrO_1.962_ and La_4_Sr_3_O_9_ as secondary phases indexed by XRD, besides Mg- and S- elements as contamination indexed by EDX analysis. As the orthorhombic phase’s Mn–O bond length is much smaller than the rhombohedral phase (Co–O and Fe–O), LSM perovskite nanoparticles may appreciably decrease effective bandgap energy with increasing orthorhombic phase (Tütüncü and Srivastava 2008). Arabi et al. established that LSM has 2 eV as a bandgap which is a main factor for enhancing photocatalytic activities (Arabi et al. 2019a, b). Morphological reason denotes to rod-like shape of LSM nanoparticles, as shown in the SEM image of Fig. 4a, that could accomplish maximum MB degradation in minimum time owing to the electron relay process (Shenoy et al. 2020; Anchan et al. 2019). Furthermore, the high degradation rate for La_0.7_Sr_0.3_MnO_3_ is more about 3.25 and 2.23, than the other samples ascribed to reduce optical bandgap energy, *E*_*g*_, which increases the free electron–hole pair (Gratzel 2001), where M–O bond length and M–O–M bond angle depend on the type of transition metal cation (Mn-Co-Fe), which plays a critical role in modifying electron bandwidth (*W*) and hence the bandgap of perovskite (Z. Zhang et al. 2010). The empirical formula relating *W* with bond length and angle is *W* α cos θ/*d*^3.5^_Mn-O_, where θ is ½ [π–(Mn–O-Mn)] and d_Mn–O_ is the M–O bond length (P.G. Radaelli et al. 1997). The bandgap relates to *W* as follows: *E*_*g*_ = *Δ-W*, where Δ is the charge–transfer energy)M. Medardeet al. 1995). LSM perovskite nanoparticles may appreciably increase *W*’s value and decrease effective bandgap energy. In addition, the 3*d* conduction band edge of Mn^4+^ (*E*_cb_ =  − 5.83 eV) is lower than Fe^3+^ (*E*_cb_ =  − 4.78 eV) state. It brings holes in the *d* band, which may reduce the effective energy gap between the O 2p valence band and the Mn-3d conduction band, decreasing the optical bandgap width of LSM nanoparticles (Hasan et al. 2016). On the contrary, Co^4+^ in the LSC sample is unstable, where oxygen can be released, leading to oxygen vacancies and a decrease in catalytic activity (Pena and Fierro 2001a, b).

In general, the magnetic properties of perovskite NPs, such as saturation magnetization (M_S_) and coercivity (H_C_), are strongly affected by four main factors, including finite size effects, surface effects, magnetic anisotropy, and degree of crystallinity besides size and shape of nanoparticles (Nguyen et al. 2021). Ferromagnetic behavior was observed at room temperature due to nanosized particle formation (Hannora and Hanna 2019), while nano wire morphology of La_0.7_Sr_0.3_MnO_3_ sample with bandgap energy of 2 eV introduces 99% photodegradation under UVC- lights, which is confirmed by Arabi et al. (2019a, b).

### Radical trapping study and photodegradation mechanism

**Fig. 10:**
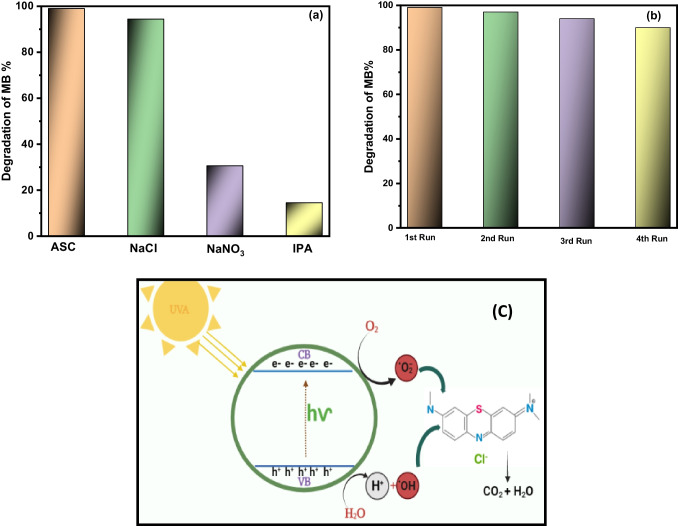
**a** Scavenger of MB dye and **b** reusability runs of MB with LSM. **c** Photocatalytic mechanism using LSM

The generation of reactive oxygen species (ROS) was detected with scavenger’s ascorbic acid (ASC), sodium chloride (NaCl), sodium nitrate (NaNO_3_), and isopropyl alcohol (IPA) for the best sample LSM to understand which type of oxidizing agent mostly promotes the degradation of MB dye under UVC- irradiation. It is well known that ASC, NaCl, NaNO_3_, and IPA are scavengers for superoxide anion (O_2_^•**−**^), holes (h^+^), electrons (e^−^), and hydroxyl radical (^•^OH) radicals, respectively (Hassan et al. 2022, Hussien and Yahis 2021). Figure 10 shows how the values of MB degradation percentage decrease with NaNO_3_ from 99 to 30.6% while using IPA degradation decreases to 14.5% after 30 min. This trend indicates that the main oxidizing agent participating in the degradation of MB is the ^•^OH radical.

8 $$\mathrm{LSM}+\mathrm{hv}\rightarrow\mathrm{LSM}\;\left(\mathrm h_{\mathrm{VB}}^++\mathrm e_{\mathrm{CB}}^-\right)$$

9 $${H}_{2}O\to {H}^{+}+OH^{-}$$

10 $${\mathrm{OH}}^{-}+{\mathrm{h}}_{\mathrm{VB}}^{+}\to {}^{\bullet }\mathrm{OH}$$

11 $${\mathrm{e}}_{\mathrm{CB}}^{-}+{\mathrm{O}}_{2}\to {\mathrm{O}}_{2}^{\bullet -}$$

12 $${\mathrm{O}}_{2}^{\bullet -}+{\mathrm{H}}^{+}\to {\mathrm{HO}}_{2}^{\bullet }$$

13 $$2\;\mathrm{HO}_2^\bullet\rightarrow2\;{\mathrm H}_2{\mathrm O}_2+{\mathrm O}_2$$

14 $${\mathrm H}_2{\mathrm O}_2\rightarrow2\;{}^\bullet\mathrm{OH}$$

15 $$\mathrm{MB}+{}^\bullet\mathrm H\rightarrow\mathrm d\mathrm e\mathrm g\mathrm r\mathrm a\mathrm d\mathrm e\mathrm d\;\mathrm p\mathrm r\mathrm o\mathrm d\mathrm u\mathrm c\mathrm t+{\mathrm H}_2\mathrm O+{\mathrm{CO}}_2$$

### Recycle and stability

The reusability or recyclability of the LSM material is an essential factor to assess since it delivers a detailed insight into the proposed system’s real-time implementation. A cycling experiment was performed to analyze the chemical stability and effective reusability of LSM material. Here, four successive cycles of constant photocatalytic MB degradation were performed. Each cycle involved extracting, rinsing, centrifuging, and drying the LSM catalyst. The degradation percentage of the LSM catalyst after various cycles is reported in Fig. 10c. The LSM catalyst displayed acceptable activity after four cycles, but its photodegradation performance was slightly decreased. Particularly, even after the four cycles, there was only a 9% decrease in degradation, so the possible application of LSM photocatalyst.

## Conclusion

A different route was prepared for La_0.7_Sr_0.3_MO_3_ perovskite compositions. The perovskite phase ratio is 100% by the sol–gel/auto combustion technique compared to 15% and 47% by co-precipitation. The crystallite size is between 32.3 and 44.2 nm. The particle size is between 214.5 and 174.2 nm. The sample porosity, *P*, and SSA are calculated to be 30, 40, and 22% and 4.25, 4.72, and 5.82 for LSM, LSC, and LSF, respectively. The FCE mechanism interprets a ferromagnetic behavior at room temperature in three samples. Among the prepared samples, the Fe- sample has shown the highest saturation magnetization of 0.4301 emu/g and better coercivity of 2905.4 G. The boost in the magnetic of La_0.7_Sr_0.3_FeO_3_ opens the door for its usage in a wide range of multifunctional devices. The photocatalytic decomposition of MB has also been investigated under the effect of UVC lights. The optimization process parameters were presented, such as the type and dye concentration. The percentage degradation of LSM, LSC, and LSF for 30 min is 99%, 78%, and 95.5%, respectively. The degradation rate* k* is 0.179, 0.055, and 0.08 min^−1^ for LSM, LSC, and LSF.

## Data Availability

The datasets used and/or analyzed during the current study are available from the corresponding author upon reasonable request.
